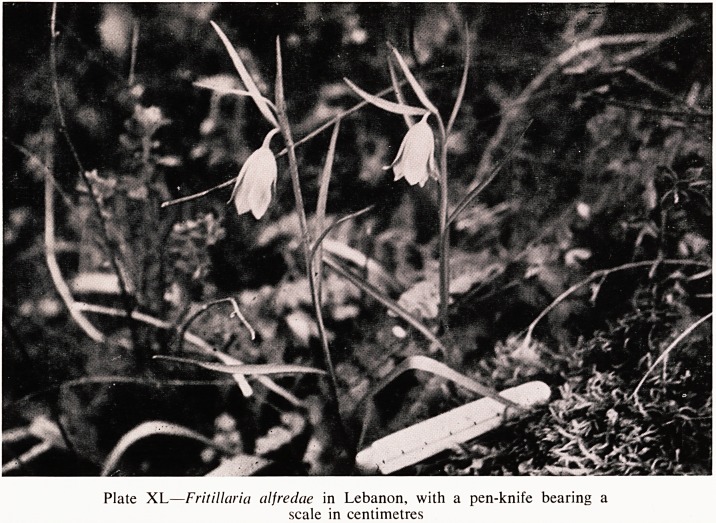# The Collecting of Wild Plants with Special Reference to Those of Medicinal or Toxicological Importance


**Published:** 1967-12

**Authors:** T. F. Hewer


### THE COLLECTING OF WILD PLANTS, WITH SPECIAL REFERENCE TO THOSE OF MEDICINAL OR TOXICOLOGICAL IMPORTANCE*

BY

T. F. HEWER

It has become customary for the President in his address to deal with a subject of interest to him as a hobby related, perhaps remotely, to medicine. I hope to persuade you of the pleasure that can be derived from an interest in wild plants, especially when travelling abroad.

The first step in acquiring such an interest is to procure a book describing the flowers to be found in some place where one is going for a holiday. Such a one is "Flowers of the Mediterranean" by Polunin and Huxley. I choose this because so many people make for that part of Europe and it is a wonderful area in which to start. Armed with such an aid to identification you may feel it worth while photographing your finds and so making a pictorial collection of them.

The next stage in the development of this hobby is the wish to bring some of the plants home. This is a very critical moment because difficulties arise. Flowers that are picked and pressed are perfectly simple but rather boring. Living plants are confiscated by H.M. Customs because their introduction is prohibited. Though this sounds like brutal bureaucracy it is in fact wise because of the danger of bringing serious plant diseases into the country. Happily it is possible, on application to the Ministry of Agriculture, Fisheries and Food—*before* one goes abroad—to obtain a permit, and this has great prophylactic value, or so it might appear. In fact, such a permit is limited to the introduction of living plants from the country you have specified and between the dates you have given; on the back of the permit is a list of the plants, chiefly conifers, that may not, under any circumstances, be introduced; and the ones that you import must be taken to a specified place—presumably your own garden—and kept there until an inspector has called to examine them, or until three weeks have elapsed.

There is, unfortunately, another danger attached to the importation of new plants: they may prove to be appalling pests. An example is the polygonum, *Polygonum cuspidatum,* known as "Japanese knotweed". It was introduced to this country from Asia in 1825. Sir Edward Salisbury has drawn attention to a note in the magazine "The Garden" published in 1879 that referred to it as "a plant of sterling merit, now becoming quite common, and is undoubtedly one of the finest herbaceous plants in cultivation." It is now a frightful weed almost impossible to eradicate. The Long Ashton people have just published an encouraging paper on the use of a new herbicide that appears to deal with it effectively (Harper & Stott, 1966). So this is another justification for the regulation requiring plants imported under licence to be kept in a designated place for a time. Unfortunately the time is not long enough and it is really the responsibility of the importer to watch his plants carefully to see what they may do, if they are not already well known.

*A Presidential Address to the Bristol Medico-Chirurgical Society, October 11th, 1967

The next requirement is to make sure that you are permitted, by the law of the country you are visiting, to dig up plants and remove them. In Switzerland I do not believe you can remove anything; in some other countries there is a list in local Post Offices or Police Stations of the forbidden plants, and these regulations must be observed. Incidentally, there is nowhere any difficulty about collecting or introducing seeds of anything, provided you are not stealing from private property. I should emphasise that I am dealing only with wild plants, not those sold by nurserymen abroad. I am sure it is not necessary to remind anyone that rare plants must not be dug up in large numbers, or perhaps at all, for fear of exterminating them.

Let us suppose that you have decided that you want to bring some living plants home, that you have a reasonable chance of cultivating them adequately, and are prepared to take the trouble to write for a permit; how do you actually collect and transport them?

Choose always a small plant, a seedling; shake all the earth off its roots and put it in a vasculum or some such tin until you get back to your lodging. Keep a diary and make an entry describing the situation of each plant: give it a number for identification. Then put the plant in a polythene bag, that you can either bring with you or make on the spot, to the appropriate size, by cutting a segment off a roll of polythene tubing, 5 inches flat, sealing one end with scotch tape and incorporating a bit of paper with the number written on it, in the tape. The other end is to be closed with a wire twist. If the plant is very leafy and moist it may be necessary to remove some of the leaves, and, in any case, one must inspect the bag every evening, especially in warm weather, to deal with a condensation of moisture which will otherwise inevitably lead to death of the plant from botrytis. One is between the devil of botrytis and the deep sea of dehydration. Experience alone can tell, but with good luck one can keep plants alive in cool conditions for three weeks like this. I have had bitter experiences in the past and am beginning to learn how to do it. When you get them home they must be treated like cuttings in a shaded bed of peat and sand.

Bulbs are much easier. Narcissi and crocuses and the like can be dug up in full bloom, the flowers removed and the leaves left to dry off gradually. Take with you a roll of tubular gauze. Cut off an appropriate length, tie one end, with a numbered label attached; fill the stocking with the bulbs and tie it off.

Cyclamen corms are rather tricky. They should not get quite dry and must not suffer condensation. I put them in a fairly large polythene bag and keep it more or less open.

Even if you decide against going to these lengths you will find such an interest a great inspiration and stimulus to travel to out-of-the-way places. I have always preferred these to Blackpool or Monte Carlo but since I developed this interest, some twenty years ago, I have gone almost exclusively to outlandish places.

Starting with Morocco, one can fly to Marrakesh (there is not time in an N.H.S. holiday to go so far by other means), and travel cheaply by native bus. One may have to stay in primitive lodgings in places where there is no hotel in the European sense, and spend the day exploring the countryside. One can live on oranges throughout the day. This country is fascinating; it ranges from desert to rocky snow-capped mountains in the High Atlas, with gentler wooded hills in the Middle Atlas. This last area is the best for our purpose.

In Lebanon one needs to go inland, preferably by hired car, as far as Baalbek, where *Hyoscyamus niger* grows, and up to the mountains at 2,000 metres where all the alpine plants abound.

In southern Anatolia, the south coast of Asiatic Turkey, there are lovely little villages, beautiful wooded hills full of flowers, and quite unexpected, unexplored Greco-Roman remains. There are also amusing means of travelling, by camel and by donkey. Inland it gets more arid and goats continue the destruction of everything that has survived the age-long deforestation.

In Greece it is hot in the summer and if one cannot get there in the spring one has to climb the mountains to find any recognisable flowers. Mount Olympus is a wonderful site for this purpose, high up above the plain, with wonderful views for many miles (Plate XXIX).

Nearer home there is Provence. Here around Mount Lubéron there is lovely country and many botanical treasures.

If one must take one's holiday in August it is not a bad plan to go to the Arctic to look for flowers. They are not spectacular but some are well worth while. In some areas cotton grass is very effective and adds a lot to the landscape. Then there is that lovely delicate little *Linnaea borealis* (Plate XXX) and many others, in the depth of the forests.

With that introduction to the subject I should like to select for more detailed consideration some particular flowers that I find attractive for various reasons, including their alleged pharmacological value and, in some cases, their undoubted toxicological importance.

My first introduction to the subject of the medicinal value of wild plants was at the hands of a remarkable man whom I met in the mountains of New Mexico in 1928. He was an odd character and I spent some time in his company, to the advantage of my education. He was an authority, so it seemed, upon the value of a great many of the plants that we saw on our horseback journey. The odd thing was that they fell into three groups — stomachics, aphrodisiacs, and cures for gonorrhoea.

A fondness for hellebores is perhaps rather depraved because, with the exception of the well-known Christmas Rose *(Helleborus niger)*, they are not strikingly pretty and one and all are poisonous. In this country we have only, as wild plants, the green hellebore, or bear's foot, and the stinking hellebore, or setter-wort, but in southern and central Europe there are others, such as *Helleborus cyclophyllus* (Plate XXXI), and the Christmas Rose itself. I came across the latter one day last year when I was driving my Land Rover through some forest, well off the road, in north-west Jugoslavia. The ground was covered with them. They are called "niger" although their flowers are remarkably white; and this is an example of the teasing nature of botanists who found that the roots were black and so named it, at least as early as the 16th century.

Gerard, in his herbal published in 1597, described four species but preferred the black hellebore, which was then called by the Dutch the "Christwurtz" because it flowered at Christmas. He recognised that it was dangerous and advised that its use should be restricted to "robustious and strong bodies"!

But he did prescribe it for "mad and furious men...... for the falling sickness, for lepers...... and briefly for all those that are troubled with black choler, and molested with melancholy". It was used extensively in the Middle Ages in the treatment of insanity. The last of several specific prescriptions that he used was to dry the leaves "in an oven, after the bread is drawne out, and the powder thereof taken in a figge or raisin...... and eaten (it) killeth wormes in children exceedingly."

Until relatively recent times a tincture of hellebore was used for intestinal worms and an infusion for killing lice. In the sixteenth century and earlier the horse-leeches or farriers—the vets of those days—used the root of the setter-wort, that is to say the stinking hellebore *(Helleborus foetidus)*, for treating diseases of the lungs in cattle. The method they employed was to cut a slit in the dewlap and insert a piece of the root. They called this "settering" and Gerard remarks that this was "a manner of rowelling as the said Horse-leeches doe their horses with horse haire twisted, or such like, and as in Surgerie we do use with silke, which in stead of the word *Seton*, a certaine Physitian called it by the name Rowell; a word very unproperly spoken of a learned man, because there would be some difference between men and beasts." A rowel was a piece of leather or other material placed beneath the skin in horses to cause discharge of humours; and a seton was what surgeons used in their human patients. Clearly the procedure was the same and I wonder whether the prefix "setter-" in setter-wort may not have been derived from "seton"; the Oxford English Dictionary says its origin is unknown. Incidentally, in some parts of Italy hellebore roots are still used by veterinarians for settering.

To come to more modern times, the poisonous principles in hellebores are two glycosides, helleborein and helleborin. The former has a digitalis-like effect on the isolated frog heart, and the latter according to Gessner has anaesthetic properties and in large doses cause severe depression of the central nervous system. This last may be the basis for what success it ever had in treating insanity.

The only paeonies that I have actually collected are *P. coriacea* in Morocco; *P. arietina* in eastern Turkey. *P. clusii* in Crete, and *P. mascula* in Portugal. All these are rather similar in appearance. *Paeonia mascula* also grows on Steep Holm, the island off Weston-super-Mare, but its origin is very doubtful. It may have been introduced by the monks who lived there in the thirteenth century. They might well have brought it with them because the roots of paeonies are credited with all sorts of medicinal virtues. In central Europe it is still popularly supposed to be a cure for gout, asthma, convulsions, tooth-ache in children, and epilepsy. Gerard in his Herball quotes Galen as saying that it is good for jaundice and for pain in the kidneys and urinary obstruction. It was also, in the 16th century in this country, believed to be a remedy for mental disorders, nightmares and epilepsy. Gessner lists some glucosides and an alkaloid, peregrinin, that can be extracted from paeony roots and seeds. The seeds cause gastroenteritis, vomiting and diarrhoea.

Another plant I want to mention is a spectacular one that comes originally from the Caucasus but is grown fairly widely in Europe in gardens, though rather daringly because of its great size and extremely prolific seeding. This is a huge cow-parsley, *Heracleum mantegazzianum* (Plate XXXII). It had never occurred to me that this was particularly unpleasant until this August when my younger son helped me in the garden by cutting them down, just in time to stop them seeding all over the place. He discovered that the stems were hollow and could be used as horns by blowing down them. Unfortunately next day he developed quite alarming blisters around his mouth and on his arms and other places where the sap of the plant had touched his skin. The skin lesions persisted for nearly three weeks and left a central pale area with a deeply pigmented margin.

On enquiry I found first that a related species, *Pastinaca sativa,* the common Wild Parsnip in this country, has just the same effect, and on one occasion laid low a whole camping party of Boy Scouts who had to clear the ground of them before pitching their tents. Gessner says that this plant has a serious effect on cattle if they eat it and that this is due to the photosensitising effect of a fluorescent furocumarin and that *Heracleum sphondylium*, another species of the genus *Heracleum*, also contains this furocumarin. In cattle that eat the leaves of these plants there are severe skin lesions, with blisters, in all parts of the skin that are not protected by hair from sunlight, and moreover sunlight coming through glass is effective in this respect so that animals have to be kept in the dark until the poison is eliminated.

Cornevin (1893), in his book that is a classic on the subject of poisonous plants, gives a fascinating account of the effect of *Heracleum sphondylium.* It appears that in August 1856 a band of workmen was employed to clear a large area of these weeds from a park in the Belgian province of Namur. Next day they developed an erysipelatoid eruption with large blisters on their wrists and, particularly, on their left arms. These persisted for up to three weeks and the men were incapacitated all that time. There was a public enquiry. It seems that the day on which they did this work was very warm and misty and the sun did not penetrate the haze until the afternoon. Some of the men started work early in the morning, when the plants were wet with dew, but some others arrived after mid-day when the mist had vanished. The latter were far less severely affected and it was concluded that some poisonous substance exuded from the plants remained in the dew but evaporated in the sun. There is an alternative explanation that I shall discuss in a moment. Incidentally, the reason why their left arms were affected was that they tore up the plants with their hands and gathered them in bundles in their left arms. The workmen gave some of the plants to cows to eat and these animals developed gastro-intestinal irritation with diarrhoea and severe thirst. It is clear that the alimentary tract troubles in the cattle were direct effects and nothing to do with photosensitisation. The plants also contain some saponins, and many saponins—that is to say, compounds of steroids with various sugars—are direct irritants, so it is possible that the furocumarins are responsible for photosensitisation if they are absorbed, and the saponins are responsible for direct local effects by contact. To test this hypothesis I was able to obtain, by the kindness of Dr. J. MacMillan, of the University Department of Chemistry, a small quantity of a mixture of the furocumarins that are present in *Heracleum,* namely sphonidin, bergapten, pimpinellin and isopimpinellin. This was provided by Mr. E. A. Baker, from the Long Ashton Research Station. I applied a spot of this alcoholic solution to the skin of my son's upper arm, and of my own, covered it with a dressing and waited 20 hours.

There was no reaction. This showed only that the simple direct action of these furocumarins on the skin was not injurious. Mr. Baker, of Long Ashton, has kindly lent me a translation of a recent excellent Russian article, by Kuznetsova, on the cumarins and furocumarins; in this it is said that the furocumarins may cause burns of the skin when applied locally in the presence of sunlight. So I got the Physiotherapy Department to give a strong erythema dose of ultra-violet light to an area on my arm where I applied some more of this solution. The result was again negative; so I don't know what is responsible for the lesions, but it is possibly a saponin, or maybe a combination of the two.

This same Russian author says that furocumarins are used for the treatment of vitiligo and that the umbellifer, *Ammi majus,* was used for this purpose by the ancient Egyptians. In the Soviet they now use a preparation called "ammifurin", obtained from *Ammi majus.* One of the furocumarins in this plant is bergapten, which is also present in *Heracleum.* In this country the dermatology textbooks mention oil of bergamot for local application to patches of vitiligo. This oil is obtained from the peel of *Citrus bergamia,* an orange that grows in Calabria. The active principle of this oil—if indeed it is active—is again the furocumarin bergapten. A lot of work is being done in France and Italy, as well as in Russia, on these photosensitising agents in the treatment of disorders of pigmentation. I have been told that the most that this oil achieves in the treatment of vitiligo is the production of a few pigmented spots. This in itself is an interesting observation because the skin lesions on my son's skin had pigmented margins after the blisters healed, but now, some two months later, the whole areas involved are definitely more pigmented than the surrounding skin. This pigmentation is patchy but quite appreciable and if this could be achieved all over an area of vitiligo it would probably be an improvement. Perhaps it is necessary to apply sufficient of the preparation containing bergapten to produce blistering of the whole surface to be treated.

This subject of the stimulation of pigmentation is of considerable theoretical as well as practical importance. Sunlight, by virtue chiefly of ultra-violet light, stimulates melanin production by the melanocytes in the normal skin. In vitiligo the melanocytes are present but inactive and it is not known what factor is lacking. It would be interesting to discover whether the furocumarin, bergapten, is capable of stimulating them to produce melanin and whether it needs the addition of sunlight. Sunlight alone certainly has no such action on vitiligo. My own brief experiments are clearly not reliable.

I should like to deal next with cyclamen. These are some of the most exciting plants that one finds around the Mediterranean. The name "cyclamen" is derived from the Greek word meaning circular, and this applies to the habit of all but one of them of coiling the stem of the flower down to the ground, like a closely wound spring, as soon as the seed capsule is formed. There are several distinct species and some subspecies and I want to show you some of them.

The first that I collected, on the top of a mountain near Florence, was *Cyclamen neapolitanum.* It flowers in the autumn and likes the shade of trees. It is the easiest of all to cultivate in shady places in gardens in this country.

Then I found *Cyclamen europaeum* in Yugoslavia, and since then in many other countries; it also blooms in the autumn and is scented.

In Yugoslavia, and in the eastern Mediterranean generally, is *Cyclamen repandum* (Plate XXXIII) that blooms in the spring.

One day in 1963 I left Syria in something of a hurry, because they were having pro-Nasser riots in Aleppo where I was, and got into Turkey through the Amanus Mountains to Iskenderun. In these mountains I saw some tiny cyclamen under some bramble bushes. They proved to be a very small form of *Cyclamen coum,* otherwise known as " orbiculatum " because their petals are arranged in a circular fashion like a propeller.

In Lebanon and southern Anatolia I have found *Cyclamen persicum,* the ancestor of all the florist's cyclamen that one gives one's aunt at Christmas. It is much taller than any of the others and the flower stalks do not coil downwards when the flowers fade. It blooms in the spring and grows in stony ground, with the corms deep down (Plate XXXIV). This means that the stones protect them from the heat of the sun in the summer but allow good drainage. This is a helpful observation for those who try to grow these plants after their first flowering—a difficult enough job!

Then there is *Cyclamen graecum* that has wonderfully intricate patterns on its leaves, and grows deeply embedded in the rock of cliffs in southern Anatolia; it is extremely difficult to collect, needing quarrying! I have never seen it in bloom in its native haunt because I have always made my expeditions at Easter, and it flowers in the autumn.

And in Crete there are two that are found nowhere else — *Cyclamen pseudograecum* (Plate XXXV), a subspecies of *C. graecum;* with the same habit of growing in holes in the rock; and the second is *Cyclamen creticum* (Plate XXXVI) that is like *repandum,* spring flowering, but white. In the Taurus Mountains of southern Anatolia I also found *Cyclamen cilicium* that blooms in the autumn.

An old name for cyclamen is sow-bread, indicating that it is eaten by pigs, but it is also eaten by mice and, I suspect, grey squirrels.

According to John Gerard in his famous Herball cyclamen root has a great many pharmacological virtues—but, then, so did most plants in those days! Among the more interesting of these are the two following prescriptions :

" The root hanged about women in their extreme travell with childe, causeth them to be delivered incontinent, and taketh away much of their paine "

and

" Being beaten and made up into trochisches, or little flat cakes, it is reported to be a good amorous medicine to make one in love, if it be inwardly taken "; on the other hand, Gerard also records some dangers :

" It is not good for women with childe to touch or take this herbe, or to come neere unto it, or stride over the same where it groweth; for the naturall attractive vertue therein contained is such, that without controversie they that attempt it in manner abovesaid, shall be delivered before their time : which danger and inconvenience to avoid, I have (about the place where it groweth in my garden) fastened sticks in the ground, and some other sticks I have fastened also crosswaies over them, least any woman should by lamentable experiment find my words to be true, by their stepping over the same."

In my own garden, oddly enough, I put sticks and string to stop people walking on them !

In fact, Gessner, the Professor of Pharmacology at Heidelberg at the present time, says that the saponin, *cyclamin,* that can be extracted from the tubers is a powerful local irritant. In man 0.3 gram by mouth of the corm of the plant causes severe gastroenteritis and larger doses in the region of 8 grams have been fatal from respiratory failure. In some animals it causes haemoglobinuria, but pigs are not affected at all. Altogether it is not to be recommended, even as an aphrodisiac. In fact, in Italy it is used for poisoning fish in rivers and in antiquity it was employed as an arrow poison. A very little put into a pond will kill all the fish.

Another peculiar family is that of the *Aristolochia,* or Dutchman's Pipe. Many of them are inconspicuous and I have collected several in various places but I should like to show you one rather spectacular one. Last Easter vacation I was climbing down a cliff in the White Mountains of Crete when I nearly stepped on an extraordinary and rather alarming thing on a narrow ledge of rock. It proved to be *Aristolochia cretica* (Plate XXXVII).

All the aristolochias are poisonous. They contain some bitter alkaloids called, collectively, aristolochine. One of them, aristolochic acid, is absorbed from the stomach and acts on the capillary endothelium, producing haemorrhages, and also renal tubular necrosis. It is an abortifacient but crude preparations of the plant have been used in small doses in the past to aid expulsion of the membranes after delivery: hence another name for the genus — " birthwort " — and the derivation of the name " aristolochia " (aristo=best). Dioscorides is quoted by Gerard as prescribing one dram weight of the plant drunk with myrrh and pepper in wine after delivery.

Arums are an interesting genus. In addition to the common Lords-and-Ladies, or Cuckoo-pint, there are some spectacular exotic species in the Mediterranean region. *Arum dioscoridis* and *Arum palaestinum* (Plate XXXVIII) are both to be found in Lebanon. They both have a horrible smell like rotten meat when they fade, but they are magnificent plants.

The root of arums is rich in starch and, in the time of Elizabeth I, when starch was used a great deal for ruffs and other items of apparel, this was a favourite source of it (Prime, 1960). Wheat at that time was scarce and its use for making starch was forbidden by Royal command. In fact the making of starch became a great monopoly given by the Queen to her favourites. The whitest starch of all was prepared from arum roots and the second best from bluebell bulbs. Unfortunately the arum roots contain calcium oxalate crystals, a glucoside, and an alkaloid of uncertain nature—aroin—and some or other of these make the starch (to use Gerard's words) " most hurtfull to the hands of the Laundresse that hath the handling of it, for it choppeth, blistereth, and maketh the hands rough and rugged, and withall smarting."

It seems that all parts of the cuckoo-pint, root, leaves, and seeds, are poisonous, being extremely irritant to the alimentary tract. On the other hand Gerard has an interesting bit of information about its use by wild bears. He

Plate XXIX—Plant collecting on Mount Olympus

Plate XXX—*Linnaea borealis* in Lapland

Plate XXXI—*Helleborus cyclophyllus* in Yugoslavia

Plate XXXII—*Heracleum mantegazzianum,* 12 ft. high

Plate XXXIII—*Cyclamen repandum* in Yugoslavia

Plate XXXIV—*Cyclamen persicum* in Lebanon

Plate XXXV—*Cyclamen pseudograecum* in Crete, showing a large corm, excavated from the rock, with its delicate stalk and the seed vessels hanging from their coiled stems

Plate XXXVI—*Cyclamen creticum* in Crete

Plate XXXVII—*Aristolochia cretica* in Crete

Plate XXXVIII—*Arum palaestinum* in Lebanon

Plate XXXIX—*Euphorbia acanthothamnos* as rounded spiny bushes in Greece, with *Phlomis fruticosa* in bloom behind

Plate XL—*Fritillaria alfredae* in Lebanon, with a pen-knife bearing a scale in centimetres says " Beares after they have lien in their dens forty daies without any manner of sustenance, but what they get with licking and sucking their own feet, doe as soone as they come forth eat the herbe Cuckow-pint, through the windie nature thereof the hungry gut is opened and made fit againe to receive sustenance, for .... it is quite shut up as Aristole, Aeliànus, Plutarch, Pliny, and others do write."

The Genus *Dracunculus* belongs to the same family and is a very sinister looking one. *Dracunculus vulgaris,* the Dragon Arum, found in Turkey, is a beautiful thing with dark spots on its stalk and branches; and *Dracunculus vulgaris var. cretica,* is a bigger and darker plant in Crete. I have no photograph of it in bloom but saw one in bud and opened it; a fearsome nearly black enormous bloom. Dioscorides and Pliny say that if the leaves are rubbed on the skin they prevent vipers from biting, and Galen used it for diseases of the lungs.

*Arisarum vulgare*—Friar's Cowl—is an attractive little plant. Gerard has little use for it but says it is reputed to heal chronic ulcers.

The spurges—*Euphorbia*—are an attractive and enormous genus of about 1,000 species, ranging from trees in Africa to little weeds in the garden but some are good and well-known garden plants, some of which I have collected abroad. *E. wulfenii* is one of the most spectacular. It abounds in Greece and Jugoslavia. *E. characias* is very like it. I have a plant that I collected from the ruined garden of the Marquis de Sade at La Coste in Provence. One of the loveliest, not hardy in this country, unfortunately, is *E. acanthothamnos*—a plant that adds so much to the landscape of Greece (Plate XXXIX). *E. dendroides,* in Crete, is a small and beautiful tree. And then there is the common Caper Spurge, *Euphorbia lathyris,* found all over Europe.

They all have one thing in common—a milky juice that exudes when any part is cut—and they are all poisonous. The active principle has not been accurately determined but they contain a triterpene called *euphorbol* that may be responsible, but there is also 7,8-dioxycumarin. Application of the juice to the skin produces blisters and may even cause gangrene of extensive areas. Two seeds of *E. lathyris,* taken by a child by mouth, have been fatal. Gessner says that the juice was used in ancient times to remove warts and freckles. In America the caper spurge is also known as the Mole Plant or, in Southern California, as the Gopher Plant, because it is reputed to drive away moles and gophers from the neighbourhood where it grows. Can this be a confusion between the moles or naevi on the skin and the little animals that burrow?

In Africa the juice of some Euphorbias is still used as an arrow poison. Gerard gives it all sorts of uses, including the application of the juice, with oil added, to the skin for the treatment of sciatica. He warns that if it is taken inwardly it " setteth on fire, scorcheth and fretteth, not onely the throat and mouth, but also the stomacke, liver and the rest of the entrals, and inflames the whole body." An antidote is said, by Gerard, to be provided by the juice of "Anteuphorbium " which, judging by his illustration, is a *Sedum.*

The last flowers that I want to mention are the fritillaries. There are many kinds that grow in the eastern Mediterranean countries and there is, of course, the well-known snake's head fritillary, *Fritillaria melaeagris,* that grows still in profusion in Magdalen Meadow in Oxford.

I have found five attractive ones in Asia Minor; *F. alfredae* (Plate XL) in the Lebanon, is the rarest of all; *F. libanotica,* also in the Lebanon, is much commoner but very local; *F. acmopetala* is confined to Asia Minor and I got it in southern Anatolia; *F. forbesii* I found on the Marmaris peninsula; and *F. pontica* is fairly widespread in Greece as well as Turkey.

Some, if not all, fritillaries contain about 0.1% dry weight of a thermolabile alkaloid, imperialin, that is a myocardial poison causing cardiac arrest, according to Gessner. Henry (1949) gives a description of eight other alkaloids found in fritillaries. They are clearly not to be eaten! Gerard knew nothing of this and is content with saying that they are "greatly esteamed for the beautifying of our gardens, and the bosoms of the beautifull."

There are occupational hazards whatever one does, and although it is clear that plant hunting is beset with dangers, especially for those who are pregnant, I hope you may feel that there is something to recommend it.

### BIBLIOGRAPHY

Cornevin, Ch. "*Des Plantes Vénéneuses et des empoisonnements qu'elles déterminent*", Paris, 1893.

Forsyth, A. A. "*British Poisonous Plants*", Ministry of Agriculture Bulletin No. 161, H.M.S.O., 1954.

Gerard, J. "*The Herball or General Historie of Plantes*"; Second Edition; London, 1636.

Gessner, O. "*Die Gift-und Arzneipflanzen von Mitteleuropa*", Heidelberg, Winter, 1953.

Harper, C. W. & Stott, K. G. Long Ashton Annual Report, 1966, 268.

Henry, T. A. "*The Plant Alkaloids*", London, Churchill, 1949.

Kuznetsova, G. A. *Trudy Botan. Inst. Akad. Nauk.* SSSR. Series 5, (1965), (12) 3-12.

Prime, C. T. "*Lords and Ladies*", London, Collins, 1949.

Viola, Severino. "*Piante Medicinali e Velenose della flora italiana*", Instituto Geografico De Agostini, Novara, 1965.

## Figures and Tables

**Plate XXIX f1:**
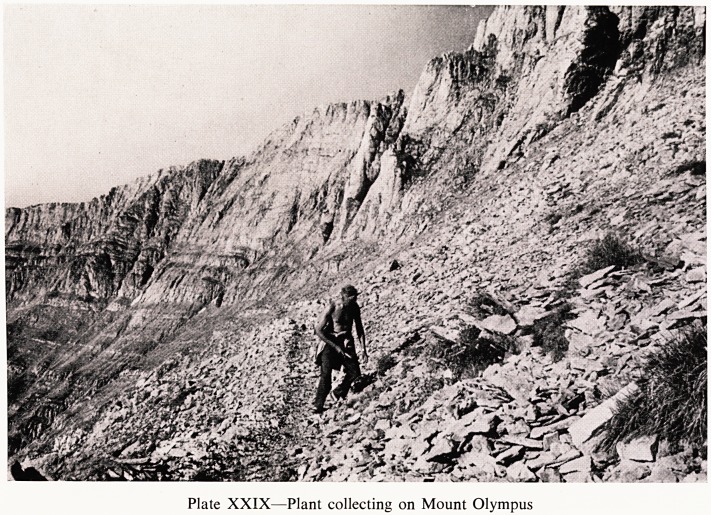


**Plate XXX f2:**
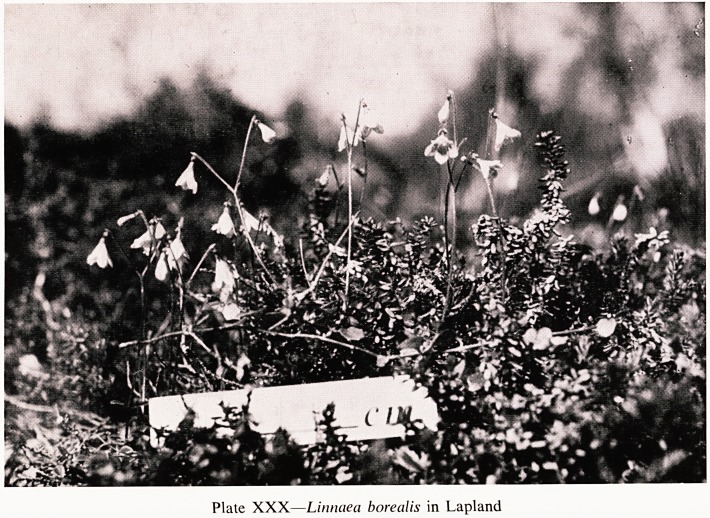


**Plate XXXI f3:**
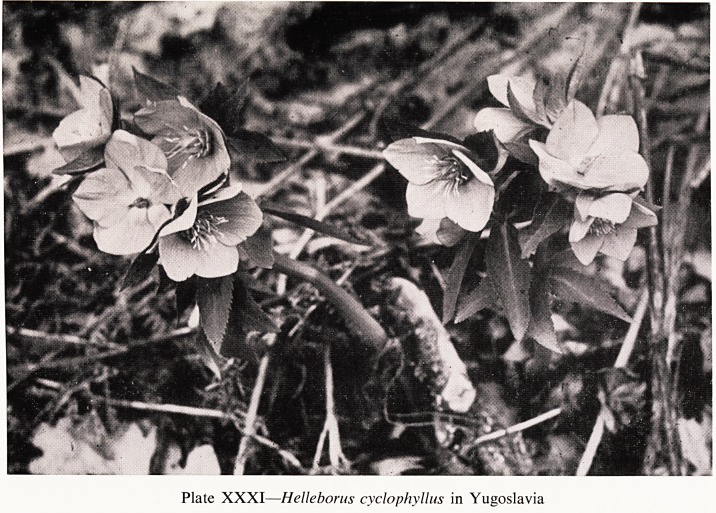


**Plate XXXII f4:**
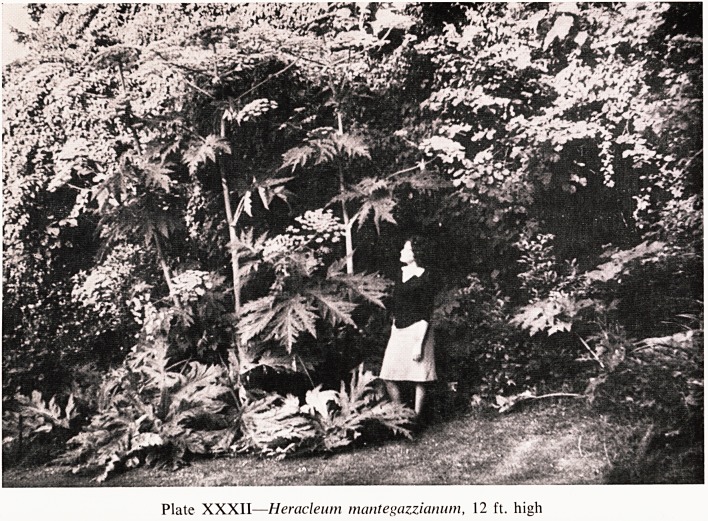


**Plate XXXIII f5:**
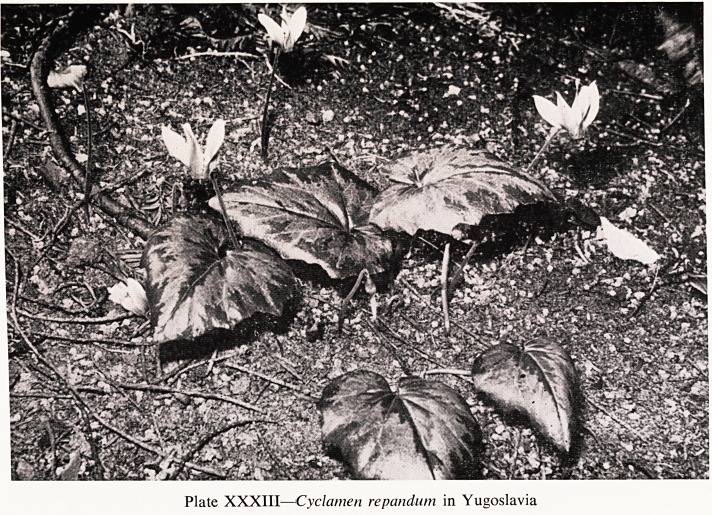


**Plate XXXIV f6:**
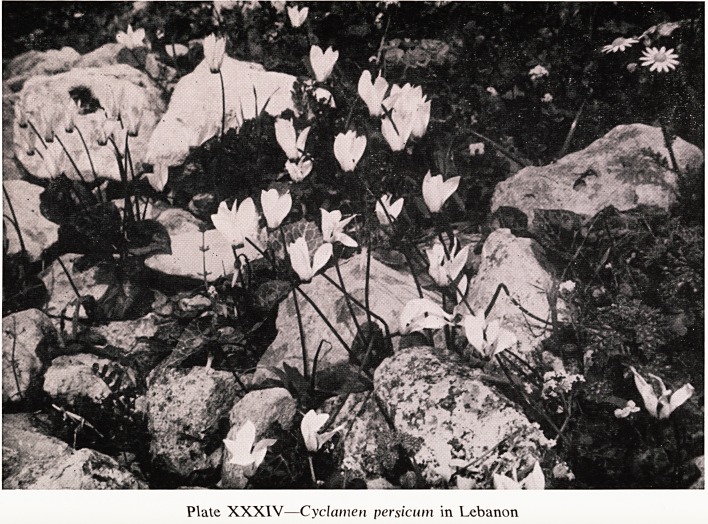


**Plate XXXV f7:**
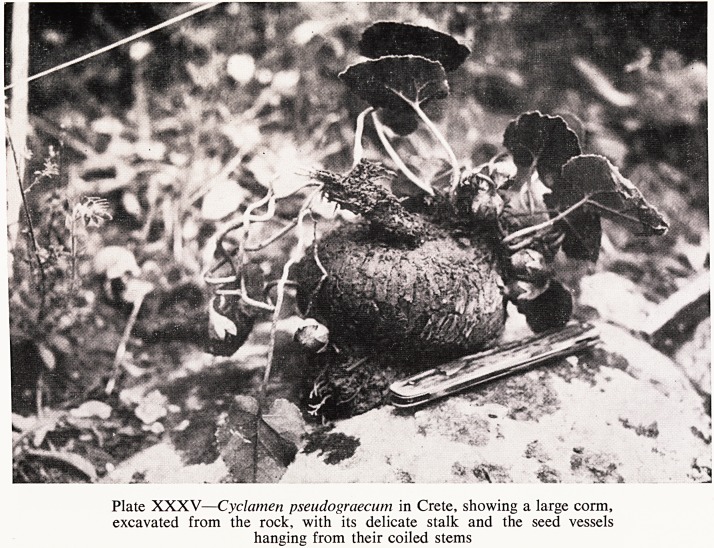


**Plate XXXVI f8:**
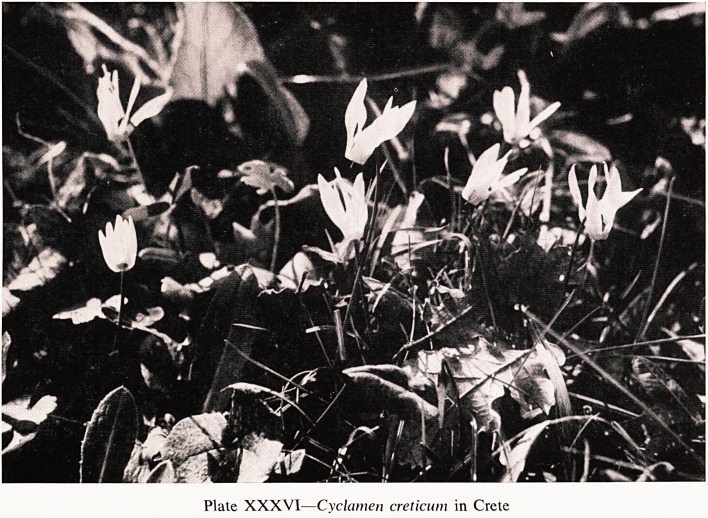


**Plate XXXVII f9:**
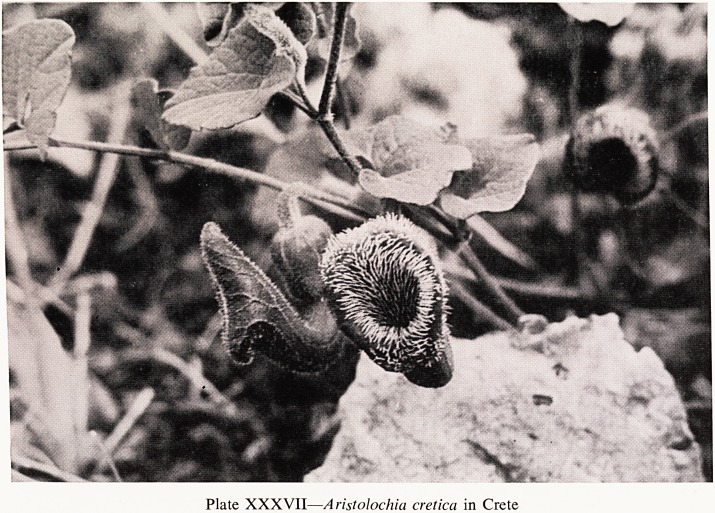


**Plate XXXVIII f10:**
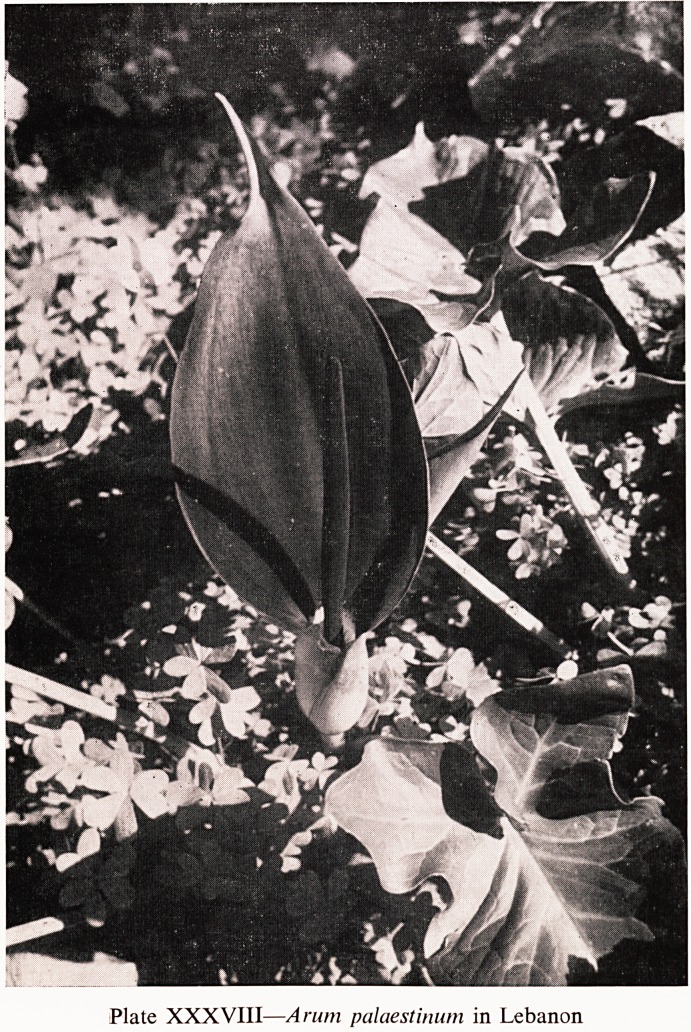


**Plate XXXIX f11:**
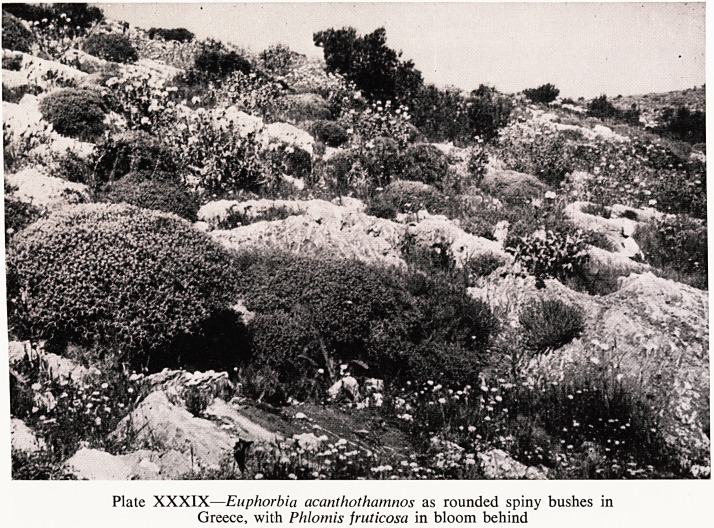


**Plate XL f12:**